# Wound Dressing Model of Human Umbilical Cord Mesenchymal Stem Cells-Alginates Complex Promotes Skin Wound Healing by Paracrine Signaling

**DOI:** 10.1155/2016/3269267

**Published:** 2015-12-31

**Authors:** Song Wang, Huachao Yang, Zhenrui Tang, Gang Long, Wen Huang

**Affiliations:** Department of Vascular Surgery, The First Affiliated Hospital of Chongqing Medical University, Chongqing 400016, China

## Abstract

*Purpose*. To probe growth characteristics of human umbilical cord mesenchymal stem cells (hUCMSCs) cultured with alginate gel scaffolds, and to explore feasibility of wound dressing model of hUCMSCs-alginates compound. *Methods*. hUCMSCs were isolated, cultured, and identified in vitro. Then cells were cultivated in 100 mM calcium alginate gel, and the capacity of proliferation and migration and the expression of vascular endothelial growth factors (VEGF) were investigated regularly. Wound dressing model of hUCMSCs-alginate gel mix was transplanted into Balb/c mice skin defects. Wound healing rate and immunohistochemistry were examined. *Results*. hUCMSCs grew well but with little migration ability in the alginate gel. Compared with control group, a significantly larger cell number and more VEGF expression were shown in the gel group after culturing for 3–6 days (*P* < 0.05). In addition, a faster skin wound healing rate with more neovascularization was observed in the hUCMSCs-alginate gel group than in control groups at 15th day after surgery (*P* < 0.05). *Conclusion*. hUCMSCs can proliferate well and express massive VEGF in calcium alginate gel porous scaffolds. Wound dressing model of hUCMSCs-alginate gel mix can promote wound healing through paracrine signaling.

## 1. Introduction

Trauma especially the extensive and nonhealing wound has gradually become a prevalent and costly problem affecting human health. It usually refers to the normal tissue suffering from structural destruction, resulting in its functional impairment [1]. Trauma initiates a series of inflammatory responses, and the surrounding tissues secrete and express large amounts of cytokines that exert chemotaxis to inflammatory cells [2]. In particular the VEGF specifically act on the vascular endothelial cells for proliferation, migration, and tube formation, prompting capillary permeability and granulation tissue formation for wound repair.

Rapid wound healing is closely related to immediate debridement, topical covering, and wound care after injury, as well as good local blood supply. A warm and moist microenvironment promotes wound healing [3]. Thus an ideal wound dressing should have a good permeability, adhesion, water absorption, and a certain degree of corrosion resistance and biodegradability [4]. A wide variety of wound dressings are in use, including the alginate, polyurethane, collagen, chitosan, pectin, and hyaluronic acid [5]. Among them, alginate is a natural polymer with a good biocompatibility that can combine with Ca^2+^ to form calcium alginate hydrogel without damage to tissues or cells [6]. This hydrogel system provides cells with extracellular matrix (ECM) analogs such as mucopolysaccharides and adhesion structure that are similar in vivo [7, 8]. It can simulate the microenvironment in vivo with greatest extent and thus may be a good cell scaffold. In addition, calcium alginate gel is almost transparent that is conducive to wound monitoring and control. Alginate has so many advantages that therefore has been widely used as wound dressings [9–11] and drug transporters [12–14].

hUCMSCs present in Wharton' jelly of human umbilical cord are adult stem cells with a multiple differentiation and self-renewal potential. Compared with other tissue-derived stem cells, umbilical cord is easily acquired, with little immunogenicity and less problems of bioethics [15]. hUCMSCs can secrete VEGF and other important growth factors necessary for angiogenesis and have been successfully applied to the treatment for trauma such as acute and chronic skin wound [16, 17]. But to date, little about hUCMSCs-alginate gel compound used in wound dressing is reported.

In this study, hUCMSCs were cultured in calcium alginate hydrogel porous scaffold. The wound dressing of hUCMSCs-alginate mix was transplanted in the excisional full-thickness skin murine models. We hypothesized that the wound dressing model of hUCMSCs-alginate gel could promote skin wound healing.

## 2. Materials and Methods

### 2.1. Materials

#### 2.1.1. Reagents and Instruments

The reagents were as follows: Fetal Bovine Serum (FBS) and Dulbecco's Modified Eagle Medium (DMEM-F12; Gibco, USA); trypsin (Hyclone, USA); osteogenic and adipogenic kit (Cyagen, USA); mouse anti-human Ig-G of CD29-PE, CD44-PE, CD45-PE, and CD105-PE antibodies (BD Biosciences, USA); oil red O, alizarin red, hematoxylin-eosin (HE), sodium alginate, anhydrous CaCl_2_, and sodium citrate (Sigma, USA); rabbit anti-mouse VEGF and CD31 monoclonal antibody and immunohistochemical kit (Boster, China).

The instruments were as follows: Cell incubator and automatic microplate reader (Thermo, USA), ultrapure water preparation instrument (Millipore, USA), high-speed centrifuge and flow cytometry (Beckman, USA), disposable plastic equipment and blood cell counts (Coning, USA), inverted phase contrast microscope and fluorescence microscope (Olympus, Japan), and so forth.

#### 2.1.2. Ethics Statement

This study was performed in strict accordance with the recommendations in the Guide for the Care and Use of Laboratory Animals of the National Institutes of Health. Umbilical cords were derived from healthy full-term male newborns with cesarean section, and informed consents were signed by their parents. Experimental animals employed herein were approved and obtained from the Ethics Committee of Animal Experiments of Chongqing Medical University (permit number: CQLA-2012-0466). All efforts were made to minimize animal suffering and the number of mice utilized.

### 2.2. Methods

#### 2.2.1. Cells Isolation and Cultivation

Umbilical cord with 3-4 cm of length was prepared, and the following steps were completed in 5 hours under sterile conditions: washed with 0.01 M PBS to remove the remaining blood; stripped the outer membrane, arteries, and veins; cut into about 1 mm × 1 mm × 1 mm tissue fragments; transferred to the bottom of disposable culture flasks prewetted with 10% FBS complete media (DMEM-F12 medium supplemented with 10% FBS), at a density of 3 fragments/cm^2^; inverted and incubated at 37°C in a humidified atmosphere with 5% CO_2_ to fix for 1-2 hours; submerged the fragments with a proper volume of 10% FBS complete media; and placed in the former incubation box to continue to culture.

Media were changed every three days. Cells were trypsinized with 0.25% (w/v) trypsin and 0.02% (w/v) EDTA when they reached >80% confluence and subcultured at a density of 2 × 10^4^ cells/cm^2^.

#### 2.2.2. Cells Observation and Identification

Cells growth and morphological characteristics were observed under an inverted phase contrast microscope and captured with Sony SLT-A77 M digital camera (Sony, Japan). Fusiform cells were acquired from the adherent umbilical cord fragments after 5–7 days. The cells could be induced to osteogenic, adipogenic, and chondrogenic differentiation. Cell surface markers were examined through flow cytometry, which exhibited that cells were positive expression for human stromal cells markers CD29, CD44, and CD105 but negative expression for hematopoietic stem cell marker CD45. These results suggested that the cells accord with hUCMSCs features.

#### 2.2.3. Cells Cocultured with Calcium Alginate Gel Scaffold

1 mL cell suspension containing 2 × 10^5^ fourth passage (P4) cells was sufficiently mixed with 2 mL 150 mM sterile sodium alginate solution (PH 7.4, and actually the final concentration of sodium alginate is 100 mM) in 6-well plates. Cells-alginate compound was solidified with 3 mL 150 mM sterile CaCl_2_ solution (PH 7.4) for 2-3 min and washed with serum-free medium for a couple of times, then a hUCMSCs-alginate gel with a bottom area of about 9 cm^2^ and a height of about 0.3 cm was acquired. For control group, an equal number of cells were cultured in another 6-well plate but without alginate-treated. Both cells in the traditional two-dimensional (2D) culture system and cells in the three-dimensional (3D) alginate gel system were cultivated with 10% FBS complete media and incubated in a same 37°C atmosphere. Media was changed every three days, and cells supernatant of each well were separately collected for further enzyme-linked immunosorbent assay (ELISA).

#### 2.2.4. VEGF Expression Test

The cells supernatant coming from last step was used to measure VEGF concentration of each well (for both 3D and 2D groups) by sandwich ELISA kits, according to the manufacturer's instructions. The test was repeated once again.

#### 2.2.5. Alginate Gel Solution and Cell Count

Equal wells of 3D cells-alginate and 2D cells were randomly selected for cell count every three days. 3D cells were acquired by following steps: cell supernatant was removed, cells-alginate gel mix was washed with PBS for several times and dissolved with 200 mM sterile sodium citrate solution (PH 7.4) in 37°C atmosphere for about 30 min, the cells mixture were centrifuged with 1000 r/min for 10 min and washed with PBS for three times, and then 3D cell clumps were acquired. Both 3D cell clumps and 2D cells were trypsinized into single cells with 0.25% trypsin and 0.02% EDTA. Cell count for both 2D and 3D cells was performed by flow cytometry. Dead cells were stained with a final concentration of 0.04% Trypan Blue and calculated under a 100x microscope, and there was no significant difference in cell death (approximately 2%) between the two groups after their digestion.

#### 2.2.6. Transwell Migration Assay

100 *μ*L suspension of 2 × 10^4^ P4 cells resuspended with serum-free cell culture medium was sufficiently mixed with 200 *μ*L 150 mM alginate in Transwell chambers. Cells-alginate compound was solidified with 150 mM CaCl2 solution for 2-3 min and washed with serum-free medium for several times. An equal number of cells without treated alginate were regarded as control group. Transwell chambers were then placed in a 24-well plate containing 700 *μ*L 10% FBS complete media and incubated in the 37°C atmosphere. Cells distribution in different gel levels (from top to bottom, with a total of about 4 layers) was observed every 24 hours and the distance between two adjacent levels was 1-2 mm. Five visual fields were randomly picked up with Sony SLT-A77Mdigital camera for cell counts in a 400x microscope.

#### 2.2.7. Full-Thickness Skin Wound Model of Mice

According to the method of Section 2.2.3, hUCMSCs-alginate gel compound was prepared and cultured with 2 mL 10% FBS complete medium. Similarly, 3 mL of 100 mM sodium alginate solution was solidified with an equal volume of 150 mM CaCl_2_ solution, and then the alginate gel was cultured with 2 mL cell supernatants solution, 10% FBS culture medium, and 0.01 mol/L PBS buffer, respectively.

**Figure 1:**
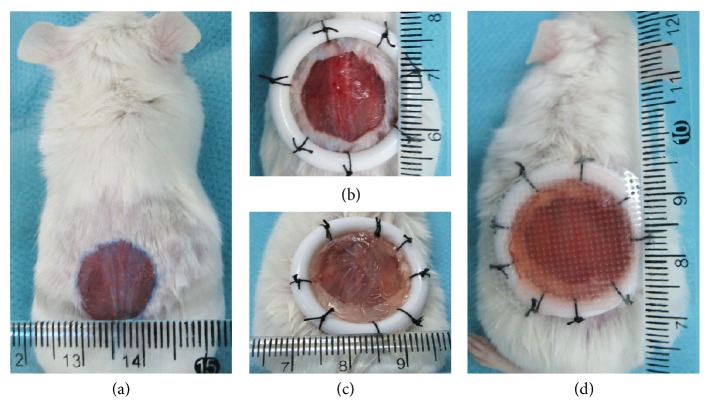
Operation steps of mice full-thickness skin wound model.

Forty male Balb/c mice (8 weeks old, weight 19–21 g) were anesthetized with 10% (w/v) chloral hydrate by intraperitoneal injection at a dose of 0.005 mL/g, and back cutaneous hair was removed. A circular full-thickness skin wound with a diameter 12–15 mm was created on the back of each mouse (see Figure 1(a)). A larger circular rigid plastic ring (inner diameter 20 mm) was used to fix the skin wound (see Figure 1(b)). The animals were randomly divided into four groups: the wounds were covered with dressing models (see Figure 1(c)) of 2 × 10^5^ hUCMSCs-alginate gel (MSC-alginate group, *n* = 10), cells supernatant of hUCMSCs-alginate gel (CS-alginate group, *n* = 10), medium of 10% FBS-alginate gel (FBS-alginate group, *n* = 10), or 0.01 mol/L PBS-alginate gel (PBS-alginate group, *n* = 10), respectively. The dimension of each gel dressing is a bottom area of about 3 cm^2^ and a height of about 0.3 cm. A transparent and breathable intravenous fluid fixture was used to fasten the gel dressings (see Figure 1(d)). All wound dressings were changed only once after three postoperative days.

#### 2.2.8. Wound Analysis

Digital pictures were taken to visualize the wounds. The remaining wounds area were calculated with software Image J (version 1.48, USA) every five days after surgery. Wound healing rate = (wound area in measurement day − initial wound area) ÷ initial wound area × 100%. Animals were sacrificed for histological analyses such as pathologic examination and immunohistochemical test at 15th day after surgery.

#### 2.2.9. Pathologic Examination

Tissue specimens were acquired from the injured skin at 15th postoperative days, soaked in 10% formalin overnight at room temperature, preserved in 70% ethanol, and embedded in paraffin. Specimens were cut lengthwise into 5 *μ*m sections and stained with hematoxylin-eosin. The stained sections were examined with an Olympus BX51 microscope (Olympus, Japan) and captured via an Olympus DP72 digital camera (Olympus, Japan).

#### 2.2.10. Immunohistochemical Test

Paraffin sections were used for immunohistochemistry tests, and the specific steps were based on a previous described method [17]. In short, the paraffin sections were dewaxed with xylene, followed by rehydration, inactivation of endogenous peroxidase, antigen retrieval, and sealing, and incubated with primary antibody (rabbit anti-mouse VEGF monoclonal antibody) and secondary antibody, respectively. Then the sections were disposed with DAB chromogen, hematoxylin redyeing, and dehydrating and sealed with neutral gum. Images of VEGF-staining cells were acquired using Olympus BX51 microscope and quantified by counting with Image J software in five random horizons in the 200 times magnification.

#### 2.2.11. Statistical Analysis

All quantitative data were given as mean ± standard deviation. All results were derived from at least two independent experiments, each of which included at least three samples. Statistical analysis was performed by the software SPSS. Statistical differences were determined by using two-factor factorial design analysis of variance, followed by Bonferroni's post hoc test at a significance level of *P* < 0.05.

## 3. Results

### 3.1. hUCMSCs Growth Characteristics

**Figure 2:**
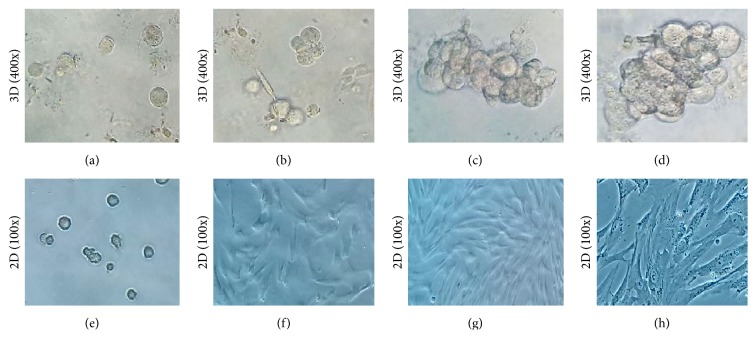
hUCMSCs cultured with alginate gel scaffold (400x). (a): for four hours, (b): for three days, (c): for six days, and (d): for nine days. (e–h): the control group in each time period (100x).

Cells began to expand after being cultivated in 3D alginate gel scaffold for 3–5 hours, with a round or oval shape and an irregular surface (see Figure 2(a)). Clone-like cell growth was observed at third day, with each cluster consisting of several cells (approximately 2–7 cells, see Figure 2(b)). Grape-like cells with tight cell-cell contact were observed in the gel at 6th and 9th days (see Figures 2(c) and 2(d)). In a word, the growth features of cells in 3D group were obviously different from those in control group (see Figures 2(e)–2(h)).

### 3.2. ELISA Analysis and Cell Counts

**Figure 3:**
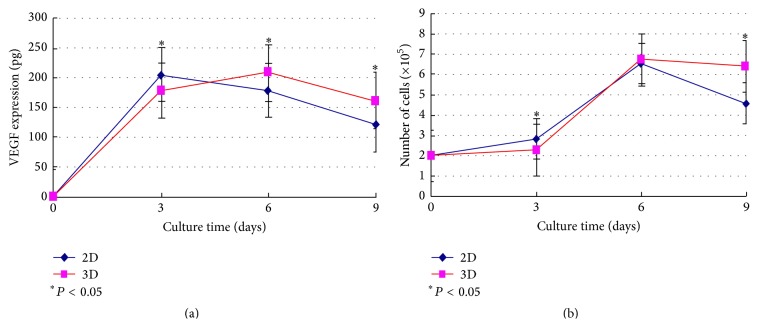
VEGF expression (a) and cell counts (b) for hUCMSCs in 3D alginate gel. 2D is the control group.

The results of ELISA analysis and cell counts were shown in Figure 3. We could find that both VEGF expression and cell number were varied in culture method and culture time (*F* = 30.28 and 20.70, resp.; *P* < 0.001). Compared to the 2D group, the number of cells and the expression of VEGF were significantly lower in the 3D group at third day, while they were significantly larger at 9th day. Cells in both groups were entering the logarithmic growth phase in 3–6 days. There was no significant difference in cell number at 6th day, when VEGF expression was significantly increased in the 3D group compared to the 2D group, whereas the tests had not been continued since massive 2D cells died after 9 days.

### 3.3. Transwell Migration Analysis

**Figure 4:**
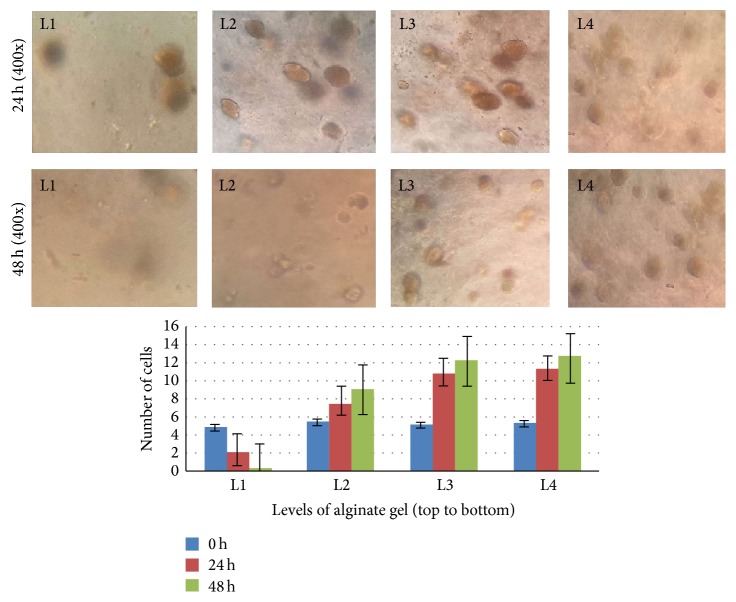
hUCMSCs migration ability in 3D alginate gel. L: the level of gel. The distance between two adjacent levels is 1-2 mm.

Migration tests showed that cells distribution was significantly uneven in different gel level after migrating for 24 hours. Cell morphology was changed from sphere to oval. The number of cells near the gel top was significantly reduced, while that close to the middle and lower levels was increased (see Figure 4). However, no cells migrated out from the gel to the bottom membrane of the chambers (total observation for 7 days).

### 3.4. Wound Healing Rate and the Pathological Evaluation

**Figure 5:**
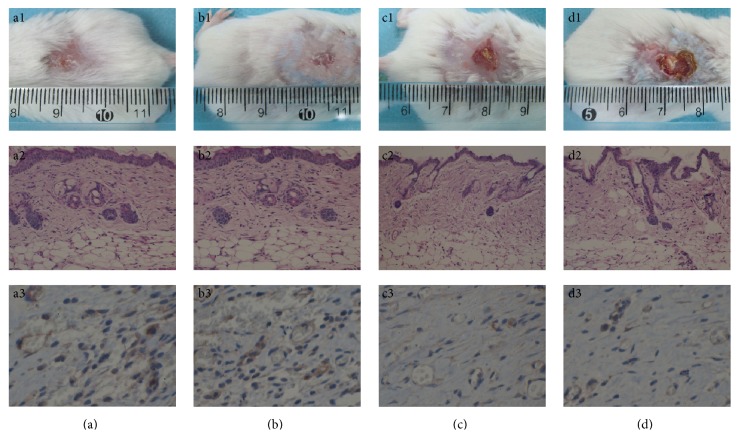
Mice cutaneous wound healing evaluation. (a): for MSC-alginate group, (b): for CS-alginate group, (c): for FBS-alginate group, and (d): for PBS-alginate group. (a1–d1): the skin wounds healing from appearance, (a2–d2): the pathology sections with HE staining (100x), and (a3–d3): the immunohistochemistry with VEGF-staining (200x).

Mice skin wounds were observed and evaluated for half a month after surgery. No animals died in this period, but two mice in the FBS-alginate group suffered purulent infection without particular manage. Wound healing rate in the MSC-alginate and CS-alginate groups in each time period was significantly faster than that in the FBS-alginate group or PBS-alginate group (*P* < 0.001), while there was no significant difference between the former two groups and between the latter two groups (see Table 1 and Figure 5).

The pathologic examination for tissue sections showed that a great number of squamous epithelium, fibroblasts, sebaceous glands, and blood capillary were viewed in the MSC-alginate and CS-alginate groups compared to those in the FBS-alginate or PBS-alginate groups (see Figure 5).

### 3.5. Immunohistochemistry Analysis

VEGF were widely expressed in the dermis cells and ECM in the new skin, especially in the renascent vascular endothelial cells. The number of VEGF-staining cells in both MSC-alginate and CS-alginate groups was significantly larger than that in the FBS-alginate or PBS-alginate groups (*P* < 0.05, see Figure 5); while no significant difference was shown between the former two groups and between the latter two groups.

## 4. Discussion

Separating cells from living tissue, expanding these cells in vitro, and encapsulating them in a 3D scaffold to repair damaged tissue are the basic strategy of modern reconstruction medicine and tissue engineering [18]. Therefore it is very important to find a suitable cell seed and relevant 3D scaffold for wound repairing.

In a liquid condition, alginate can cross-link with Ca^2+^ into calcium alginate hydrogel that is nontoxic to cells/tissues and has a certain degree of biodegradability [19, 20]. It is reported [21] that the calcium alginate gel scaffold prepared with a concentration of 100–150 mM sodium alginate is optimum for cells adhesion, proliferation, and expression of ECM, since the pore size of gel in this concentration range is uniform (50–200 *μ*m) and has a good mechanical strength. In this study, hUCMSCs grew well with a manner of grape or clone, and proliferated up to two weeks in 100 mM alginate gel scaffold, which was obviously different from the cells cultured by 2D method (see Figure 2). These results suggest that hUCMSCs maintained their growth activity in the 3D alginate porous scaffolds and could even proliferate for a longer time than in the 2D culture system in the condition of without enzymic digestion.

Trauma is a common surgical disease, and thus helping wound healing is a focus concern of a surgeon. Wound healing involves a series of overlapping and interrelated periodic pathological alterations. Many cells and ECM components participate in the repair or reconstruction of damaged tissue [22]. In the tissue engineering, stem cell therapy is often used in extensive or refractory skin wounds (including both acute and chronic wounds). There are mainly two mechanisms of stem cell therapy: a theory of cell-replacement [23], which means that the impaired host cells or tissues were structurally and/or functionally replaced with stem cells by directive differentiation; a paracrine theory [17], which means that the stem cells activate specific signaling pathway by secreting important cytokines to accelerate wound healing. However, the exact mechanism is still controversial. At present, previous studies were biased in favor of the paracrine theory and thought that the VEGF plays a critical role in wound healing process [24].

Our results showed that the number of cells and VEGF expression in 3D group were significantly less than those in the control group in first three days (see Figure 3). One possible reason is that the hUCMSCs cultured in alginate gel were in the adaptation phase and growing slowly in the other day; therefore VEGF expression in the 3D group is relatively reduced. On the other hand, some cell supernatant was retained in the gel porous scaffold and lots of VEGF failed to be detected by ELISA test, which resulted in a significant reduction of VEGF expression in the 3D group. As incubation time increases, VEGF expression at 6th and 9th days was significantly larger in the 3D group compared to 2D group, though the number of cells at 6th day did not differ significantly. Six days later, a large number of 2D cells were dead gradually, while there was no significant reduction in the 3D group. Therefore cells proliferation is hindered in 2D condition due to confluency while in 3D system they keep growing because there is still available “volume.” These results suggest that 3D culture method is more suitable for hUCMSCs to proliferate and express cytokines [25].

Transwell migration assay showed, with the extension of incubation time, that the number of cells in the upper layers of the gel reduced gradually, while it was significantly increased in the bottom layers of the gel where they were closer to the culture media (see Figure 4). However, hUCMSCs could not migrate out from the 3D alginate gel during seven days of observation. These results prompt that although the gel scaffold can provide a suitable aperture or space for cells growth and proliferation, it has no large enough channels to allow the cells to move or shuttle freely. Thus the increased cell number in the bottom layers of the gel may be attributed to the proliferation of the local cells but not the migration from the upper layers. This diffusion limitation of cells is probably determined by the concentration or stiffness of the alginate gel scaffold [26, 27]. In fact, nonetheless, the cells grew well in the gel porous scaffold, which indicates that the gel has a good permeability, and the nutrients such as oxygen and cytokines in the liquid can straightway diffuse into cells through the gel system [28]. As well, the pore size of gel at the concentration of 100 mM is enough for cells growth, combined with lots of little channels between the adjacent pores [21], which would not result in local regions of hypoxia or metabolite limitations.

Further animal experiments showed that both MSC-alginate gel and CS-alginate gel could significantly promote skin wound healing in mice, while no significant difference was found in this two groups (see Table 1 and Figure 5). Simultaneously, taking into account the fact that cells could not migrate out from the gel to the wound, we thus believe that a paracrine mechanism of hUCMSCs should explain this faster wound healing [17]. According to previously published articles [3, 29], a warm and moist microenvironment promotes wound healing. In this study, hUCMSCs-alginates mixture was transplanted into the mice skin defect, which not only had a protective effect on wounds but also kept the local wounds moist. Meanwhile, in the early stage of wound healing, the wound exudates or blood around the gel is a good medium for hUCMSCs that can continuously repair or reconstruct the wounds by expressing some important cell growth factors and promote rapid wound healing. Besides, calcium alginate gel is almost transparent that is conducive to wound monitoring and control.

In conclusion, hUCMSCs can proliferate for a relatively long time in the 3D alginate gel system and can durably secrete and express VEGF that is necessary for wound healing. This study provides feasibility for 3D alginate culture of hUCMSCs, and what is more important, it provides a theoretical basis for clinical application of hUCMSCs-alginates dressing model.

## Figures and Tables

**Table 1:** Mice wound healing rate in each group at different time after surgery.

Wound dressings (groups)	Wound healing rate (%, $\overset{-}{x}\pm s$)			Statistics (*F*, *P* value)
	5th day	10th day	15th day	
MSC-gel group (*n* = 10)	47.61 ± 2.82^▲^	80.70 ± 4.30^△^	96.60 ± 2.36^△▲^	*F* = 585.549, *P* = 0.000

CS-gel group (*n* = 10)	46.30 ± 2.73^▲^	78.64 ± 3.43^△^	94.75 ± 2.76^△▲^	*F* = 679.018, *P* = 0.000

FBS-gel group (*n* = 10)	38.21 ± 8.11^*∗*#▲^	58.22 ± 7.24^*∗*#△^	81.42 ± 4.74^*∗*#△▲^	*F* = 99.797, *P* = 0.000

PBS-gel group (*n* = 10)	37.03 ± 2.68^*∗*#▲^	56.18 ± 5.29^*∗*#△^	79.67 ± 5.76^*∗*#△▲^	*F* = 200.150, *P* = 0.000

Statistics (*F*, *P*-value)	*F* = 13.392, *P* = 0.000	*F* = 61.382, *P* = 0.000	*F* = 44.925, *P* = 0.000	

^*∗*^Compared with MSC-gel group, *P* < 0.05; ^#^compared with CS-gel group, *P* < 0.05; ^△^compared with the value at 5th day, *P* < 0.05; ^▲^compared with the value at 10th day, *P* < 0.05.

## References

[1] Lazarus G. S., Cooper D. M., Knighton D. R. (1994). Definitions and guidelines for assessment of wounds and evaluation of healing. *Wound Repair and Regeneration*.

[2] Buch S. (2014). Growth factor signaling: implications for disease & therapeutics. *Journal of Neuroimmune Pharmacology*.

[3] Triller C., Huljev D., Rucigaj T. P. (2013). Modern wound dressings. *Acta Medica Croatica*.

[4] Balakrishnan B., Mohanty M., Umashankar P. R., Jayakrishnan A. (2005). Evaluation of an in situ forming hydrogel wound dressing based on oxidized alginate and gelatin. *Biomaterials*.

[5] Boateng J. S., Matthews K. H., Stevens H. N. E., Eccleston G. M. (2008). Wound healing dressings and drug delivery systems: a review. *Journal of Pharmaceutical Sciences*.

[6] Augst A. D., Kong H. J., Mooney D. J. (2006). Alginate hydrogels as biomaterials. *Macromolecular Bioscience*.

[7] Raof N. A., Raja W. K., Castracane J., Xie Y. (2011). Bioengineering embryonic stem cell microenvironments for exploring inhibitory effects on metastatic breast cancer cells. *Biomaterials*.

[8] Wang L., Shelton R. M., Cooper P. R., Lawson M., Triffitt J. T., Barralet J. E. (2003). Evaluation of sodium alginate for bone marrow cell tissue engineering. *Biomaterials*.

[9] Thu H.-E., Zulfakar M. H., Ng S.-F. (2012). Alginate based bilayer hydrocolloid films as potential slow-release modern wound dressing. *International Journal of Pharmaceutics*.

[10] Poor A. E., Ercan U. K., Yost A., Brooks A. D., Joshi S. G. (2014). Control of multi-drug-resistant pathogens with non-thermal-plasma-treated alginate wound dressing. *Surgical Infections*.

[11] Coşkun G., Karaca E., Ozyurtlu M., Özbek S., Yermezler A., Çavuşoğlu I. (2014). Histological evaluation of wound healing performance of electrospun poly(vinyl alcohol)/sodium alginate as wound dressing in vivo. *Bio-Medical Materials and Engineering*.

[12] Kuijpers A. J., van Wachem P. B., van Luyn M. J. A. (2000). In vivo and in vitro release of lysozyme from cross-linked gelatin hydrogels: a model system for the delivery of antibacterial proteins from prosthetic heart valves. *Journal of Controlled Release*.

[13] Bhunchu S., Rojsitthisak P. (2014). Biopolymeric alginate-chitosan nanoparticles as drug delivery carriers for cancer therapy. *Pharmazie*.

[14] Sosnik A. (2014). Alginate particles as platform for drug delivery by the oral route: state-of-the-art. *ISRN Pharmaceutics*.

[15] Seshareddy K., Troyer D., Weiss M. L. (2008). Method to isolate mesenchymal-like cells from Wharton's Jelly of umbilical cord. *Methods in Cell Biology*.

[16] Sabapathy V., Sundaram B., Sreelakshmi V. M., Mankuzhy P., Kumar S. (2014). Human Wharton's Jelly mesenchymal stem cells plasticity augments scar-free skin wound healing with hair growth. *PLoS ONE*.

[17] Arno A. I., Amini-Nik S., Blit P. H. (2014). Human Wharton's jelly mesenchymal stem cells promote skin wound healing through paracrine signaling. *Stem Cell Research and Therapy*.

[18] Langer R., Vacanti J. P. (1993). Tissue engineering. *Science*.

[19] Magyar J. P., Nemir M., Ehler E., Suter N., Perriard J.-C., Eppenberger H. M. (2001). Mass production of embryoid bodies in microbeads. *Annals of the New York Academy of Sciences*.

[20] Oca-Cossio J., Simpson N. E., Han Z., Stacpoole P. W., Constantinidis I. (2005). Effects of alginate encapsulation on mitochondrial activity. *Journal of Materials Science: Materials in Medicine*.

[21] Liu Y., Ren L., Ji P. H., Wang Y.-J. (2012). Purification of sodium alginate and preparation of porous calcium alginate scaffolds. *Journal of South China University of Technology*.

[22] Shakespeare P. (2001). Burn wound healing and skin substitutes. *Burns*.

[23] Newman R. E., Yoo D., LeRoux M. A., Danilkovitch-Miagkova A. (2009). Treatment of inflammatory diseases with mesenchymal stem cells. *Inflammation and Allergy—Drug Targets*.

[24] Galeano M., Altavilla D., Bitto A. (2006). Recombinant human erythropoietin improves angiogenesis and wound healing in experimental burn wounds. *Critical Care Medicine*.

[25] Lai Y., Asthana A., Kisaalita W. S. (2011). Biomarkers for simplifying HTS 3D cell culture platforms for drug discovery: the case for cytokines. *Drug Discovery Today*.

[26] Pebworth M.-P., Cismas S. A., Asuri P. (2014). A novel 2.5D culture platform to investigate the role of stiffness gradients on adhesion-independent cell migration. *PLoS ONE*.

[27] Stowers R. S., Allen S. C., Suggs L. J. (2015). Dynamic phototuning of 3D hydrogel stiffness. *Proceedings of the National Academy of Sciences of the United States of America*.

[28] Yuan Y., Sin W.-Y., Xue B. (2013). Novel alginate three-dimensional static and rotating culture systems for effective ex vivo amplification of human cord blood hematopoietic stem cells and in vivo functional analysis of amplified cells in NOD/SCID mice. *Transfusion*.

[29] Winter G. D. (1995). Formation of the scab and the rate of epithelisation of superficial wounds in the skin of the young domestic pig. 1962. *Journal of Wound Care*.

